# A family presenting with multiple endocrine neoplasia type 2B: A case report

**DOI:** 10.1186/1752-1947-5-587

**Published:** 2011-12-20

**Authors:** Mahnaz Majidi, Vahid Haghpanah, Mahdi Hedayati, Patricia Khashayar, Mohammad Reza Mohajeri-Tehrani, Bagher Larijani

**Affiliations:** 1 Endocrinology & Metabolism Research Center, Tehran University of Medical Sciences, Tehran, Iran; 2 Research Institute for Endocrine Sciences, Shahid Beheshti University of Medical Sciences, Tehran, Iran

## Abstract

**Introduction:**

Multiple endocrine neoplasia 2B, a rare autosomal dominant syndrome, is characterized by early onset of medullary thyroid carcinoma, pheochromocytoma, marfanoid habitus and mucosal neuromas of the tongue, lips, inner cheeks and inner eyelids. Gangliomatosis of the gastrointestinal tract and its complications may also occur in patients with this disease.

**Case presentation:**

We present the case of a 16-year-old Persian man diagnosed as having a non-invasive form of multiple endocrine neoplasia 2B (medullary thyroid cancer, mucosal neuroma of the tongue, lips and inner eyelids). Our patient, who had a positive family history of medullary thyroid cancer, was of normal height with no signs of marfanoid habitus.

**Conclusions:**

Ophthalmological and oral manifestations of multiple endocrine neoplasia 2B, as in the case of our patient, are rare presentations of the disease; unfortunately in the case of our patient his condition had not been noted and acted upon until he presented to our department. The diagnosis in our patient's case was made only after his mother presented with the same condition. As a result, we emphasize that physicians should pay more attention to the oral and ocular signs of multiple endocrine neoplasia 2B in order to diagnose this fatal syndrome at an earlier phase.

## Introduction

Multiple endocrine neoplasia 2B (MEN2B) is a rare autosomal dominant syndrome [1-4]. It is diagnosed by early onset medullary thyroid carcinoma (MTC), pheochromocytoma, marfanoid habitus and mucosal neuromas of the tongue, lips, inner cheeks and inner eyelids.

Gangliomatosis of the gastrointestinal tract and its complications may also occur in these patients [5-7].

MTC arises from C cells (which secret calcitonin) of the thyroid gland [8,9]. The hereditary forms of MTC occur as a part of MEN2B, associated with germline mutation of the RET proto-oncogene [1,10]. The replacement of methionine with threonine (M918T) occurs in more than 95% of these patients [2,3,5].

After ruling out pheochromocytoma, prophylactic thyroidectomy should be performed before the age of six months to treat MTC in patients with MEN2B [2,4,6-8,11]. Here, we report a case of MEN2B in a patient, recognized only after his mother was found to have the same disease.

## Case presentation

A 16-year-old Persian man visited a referral hospital after his mother was diagnosed as having aggressive MTC. The 38-year-old woman, who had had a mass in the thyroid gland and cervical lymphadenopathy for six years, underwent thyroidectomy a year earlier after being diagnosed as having the disease. Her family members were then asked to visit a physician for further evaluation as the disease usually runs in the family.

Our patient had no history of the main symptoms of the disease, namely lymphadenopathy (LAP), weight loss, fever, café au lait spots, ocular problems, gastrointestinal problems (failure to thrive (FTT), abdominal pain, dysphagia, projectile vomiting, diarrhea, constipation, flatulence), thyroid nodule, hoarseness, dyspnea and cough.

On physical examination, his face was symmetric and there was no sign of high arched palate, mandibular prognathism, and flat nasal bridge. He, however, had bumpy lips and several neuromas on his upper and lower eyelids, lips and tongue, all characteristic of MEN2B. His thyroid gland and abdomen were normal, and there were no other remarkable finding in the physical examination. He had a normal height with no signs of marfanoid habitus. He had increased calcitonin and carcinoembryonic antigen (CEA) levels but tests for RET proto-oncogene on exon 10, 11 and 16 were negative.

The results of an ophthalmology examination showed several mucosal neuromas on inner eyelids and conjunctivae, prominent perilimbal conjunctival blood vessels and enlarged corneal nerves. His intra-ocular pressure (IOP) was normal. His upper gastrointestinal and small bowel series were also normal.

Pheochromocytoma was ruled out based on the laboratory test results. Our patient thereafter underwent a thyroidectomy. The results of pathological tests revealed a small (0.5cm) medullary thyroid carcinoma in right lobe, with surgical margins free of tumor.

Post-operative evaluation, including cervical ultrasound as well as cervical, thoracic and abdominal computed tomography, were normal. Our patient's calcitonin and CEA levels were then assessed periodically.

It should be noted that all the family members had signed an informed consent, providing the authors with an authorization to publish their information.

## Discussion

The majority of patients with MEN2B experience two main symptoms, MTC and pheochromocytoma. Individuals with MEN 2B show a variety of additional conditions, including a characteristic facial appearance with swollen lips, neuromas of the mucous membranes of the eye, mouth, tongue, and nasal cavity, enlarged colon, and skeletal abnormalities [11-13].

MTC in MEN2B, which usually develops early in life, is multi-centric and bilateral. Considering the high risk of early metastasis (to the liver and lungs) and the aggressive and life-threatening nature of MTC in MEN2B, it is vital to recognize the condition as soon as possible [6,7,9]. In view of the fact that our case was asymptomatic, he was diagnosed as having the disease only because his mother was a known case of aggressive MTC.

As prophylactic thyroidectomy before the age of six months is recommended in MEN2B cases presenting with MTC, our patient underwent the surgery as soon as he was diagnosed as having the disease [8].

The mutation of exon 16 of the RET proto-oncogene located on chromosome 10 is reported in 95% of MEN2 cases [9]. This comes while a few MEN2 cases (as our case) have been described to be negative for the RET mutations, suggesting that the mutation of other loci may also predispose an individual to developing the disease [14,15].

Mucosal neuromas of the tongue, lips, inner cheeks and inner eyelids are hallmark diagnostic findings in MEN2B [8,16]. Mucosal neuromas of the tongue, as reported in our patient's case, are almost pathogonomic of MEN2B in the presence of MTC [15]. Many primary care physicians, however, fail to detect these neuromas because of the scarce nature of MEN2B and their unfamiliarity with its symptoms [8].

There were no signs of marfanoid phenotype, which usually become apparent during puberty or early adolescence, in our patient's case [6].

The ophthalmological disorders accompanying with MEN2B are photophobia, ptosis, everted eyelids, neuromas of the eyelid and conjunctivae, dryness of the eye, increased intra-ocular pressure (IOP), prominent corneal nerves and enlarged perilimbal conjuctival vessels [6,12]. These manifestations are seen in 86% of patients at the time of diagnose [6,10,12]. Prominent corneal nerves, which were also noted in our patient, are characteristic of MEN2B [10-13]; it, however, may be reported in other syndromes as well and that could be the reason that our patient remained undiagnosed for many years despite having these typical features [10,11,13]. Early detection of such symptoms can prevent from the development of other life threatening complications of the syndrome [11,12,17].

Gastrointestinal symptoms such as FTT, abdominal pain, dysphagia, projectile vomiting, diarrhea, constipation, flatulence, intussusceptions, megacolon, and pseudo obstruction are considered as possible manifestations of MEN2B [9]. Based on the results of physical examination and imaging, there were no signs of these symptoms, mainly referred to as gangliomatosis of the gastrointestinal tract, in our patient. There are several reports showing numerous MEN2B cases remaining undiagnosed despite having diagnostic oral characteristics [6].

## Conclusions

The present article reports a rare case of MEN2B with ophthalmological and oral manifestations that was only diagnosed after our patient's mother was diagnosed as having the same disease. We therefore urge physicians to pay more attention to the oral and ocular signs of MEN2B, as early diagnosis can prevent further complications of this fatal disease.

## Competing interests

The authors declare that they have no competing interests.

## Consent

Written informed consent was obtained from the patient's legal guardian for publication of this case report and any accompanying images. A copy of the written consent is available for review by the Editor-in-Chief of this journal.

## Authors' contributions

MRMT visited and managed our patient; he also edited and supervised the revision process of the manuscript. MM and VH wrote the manuscript. PKH edited the manuscript. MH performed the laboratory tests, and analyzed and interpreted the results. BL was a major contributor to revising the final manuscript. All authors have read and approved the final manuscript.
